# A Comparative Evaluation of Holmium:YAG and Thulium Fiber Lasers in Urinary Stone Treatment

**DOI:** 10.7759/cureus.89491

**Published:** 2025-08-06

**Authors:** Meyada Ali, Chidi Nzekwue, Emily Moseley, Sohail Singh, Akshay Vinoo, Jirayr Ajzajian

**Affiliations:** 1 Urology, Worcestershire Acute Hospitals NHS Trust, Worcester, GBR

**Keywords:** holmium:yag laser, laser lithotripsy, stone-free rates, thulium fiber laser, urinary stone disease

## Abstract

Laser lithotripsy has transformed the management of urinary stone disease, with Holmium:YAG (Ho:YAG) long regarded as the standard of care. However, the emergence of the thulium fiber laser (TFL) has introduced a novel alternative with potential technical and clinical benefits. This review synthesizes data from randomized controlled trials and cohort studies published between 2019 and 2025 comparing Ho:YAG and TFL for urinary stone lithotripsy. A total of 13 peer-reviewed studies were included, with outcomes assessed across stone-free rates (SFRs), operative time, ablation efficiency, complication rates, and overall safety. While both lasers achieve comparable SFRs, several studies reported significantly higher clearance rates and shorter operative times with TFL. TFL was also associated with improved dusting performance, reduced retropulsion, and enhanced ergonomics due to its higher frequency and smaller fiber compatibility. Safety outcomes were similar, though some studies noted fewer bleeding events and greater fiber durability with TFL. These findings suggest that TFL may offer advantages over Ho:YAG in selected clinical settings. Although Ho:YAG remains the globally established modality, accumulating evidence supports the growing role of TFL in contemporary endourology, pending further long-term and cost-effectiveness data.

## Introduction and background

Urinary stone disease is a common and recurrent condition, affecting up to 12% of the global population and requiring repeated interventions due to a high recurrence rate. It is associated with significant morbidity, including flank pain, urinary tract infections, and potential renal impairment. The economic burden is substantial; in England, kidney stone disease accounted for approximately 90,000 hospital admissions in 2019-2020, costing over £324 million annually [1,2].

The advent of minimally invasive techniques, particularly laser lithotripsy, has transformed stone management, offering precision, reduced recovery time, and minimal morbidity. Since its introduction in the 1990s, the Holmium:YAG (Ho:YAG) laser has become the cornerstone of endourological stone treatment due to its ability to fragment all stone types and compatibility with flexible ureteroscopes [3]. Operating at a wavelength of 2,100 nm, Ho:YAG delivers pulsed energy absorbed by water, generating photothermal and photomechanical effects that break down calculi [3]. However, limitations such as higher retropulsion, reduced efficiency at low pulse energies, and the use of bulkier fibers have prompted interest in alternative technologies [4].

The thulium fiber laser (TFL), a more recent innovation, operates at 1,940 nm, a wavelength with a higher absorption coefficient in water. This enables more efficient energy transfer, finer stone dusting at lower pulse energies, and reduced retropulsion [5,6]. Additionally, TFL supports higher frequency settings and the use of ultra-thin fibers, improving irrigation dynamics, visualization, and scope deflection in narrow anatomical spaces [7-9]. These characteristics suggest potential clinical advantages, particularly in dusting techniques and challenging anatomies.

As endourological practice evolves, laser selection has become more than a matter of availability. It increasingly depends on aligning laser characteristics with stone composition, anatomy, and surgical goals. For instance, in retrograde intrarenal surgery (RIRS), TFL’s thinner fibers and superior irrigation may facilitate access to lower pole calyces and improve visibility in confined spaces [4]. In ureteric stones, reduced retropulsion with TFL helps prevent stone migration, potentially lowering the need for devices such as baskets. Conversely, Ho:YAG’s higher peak power may be advantageous for fragmenting harder stones or when simultaneous tissue cutting is required during complex ureteroscopic procedures [4]. Matching these capabilities to patient-specific anatomical and procedural demands is essential to maximize efficiency and outcomes.

This review aims to critically evaluate and summarize the current comparative evidence between Ho:YAG and TFL, considering efficacy, efficiency, safety, and practical implementation in endourological practice.

## Review

Methodology

Search Strategy and Study Selection

A systematic literature review was conducted using PubMed and the Cochrane Library to identify relevant studies published between January 2019 and March 2025. The search terms included “Thulium Fiber Laser,” “Holmium:YAG,” “lithotripsy,” “urolithiasis,” and “comparative study.” Eligible studies were peer-reviewed, published in English, and directly compared TFL and Ho:YAG lasers for the treatment of urinary tract stones. Included study designs comprised randomized controlled trials (RCTs), prospective comparative studies, and cohort studies. Case reports, narrative reviews lacking comparative data, and non-English-language articles were excluded. Reference lists of included studies and relevant reviews were manually screened to identify any additional eligible studies.

Data Extraction and Quality Assessment

Two reviewers independently screened titles, abstracts, and full texts to determine study eligibility, resolving any discrepancies by consensus. Data extracted from each study included publication details (author, year, country), study design, patient demographics, and clinical outcomes, such as stone-free rates (SFRs), operative time, laser efficiency, complication rates, and safety outcomes. In total, 81 records were identified through database searches and supplementary sources. After removing duplicates and trial design papers, 22 unique studies remained. Following title and abstract screening, four studies were excluded for not meeting inclusion criteria, typically due to in vivo design or lack of comparative data. In total, 18 full-text articles were reviewed in detail, and 13 studies were ultimately included in the final synthesis. Exclusions at this stage were primarily due to incomplete reporting of operative time, laser efficiency, fragmentation speed, or complication outcomes.

Due to differences in study designs, outcome measures, follow-up periods, and how results were reported, a formal meta-analysis was not appropriate. Instead, we used a descriptive narrative approach to explore patterns and compare the clinical and technical outcomes of TFL and Ho:YAG lasers. This method aligns with scoping review principles, which focus on thematic insights over statistical pooling. While this limits the ability to calculate combined effect sizes, it enables a thorough examination of current evidence, trends, and research gaps.

**Figure 1:**
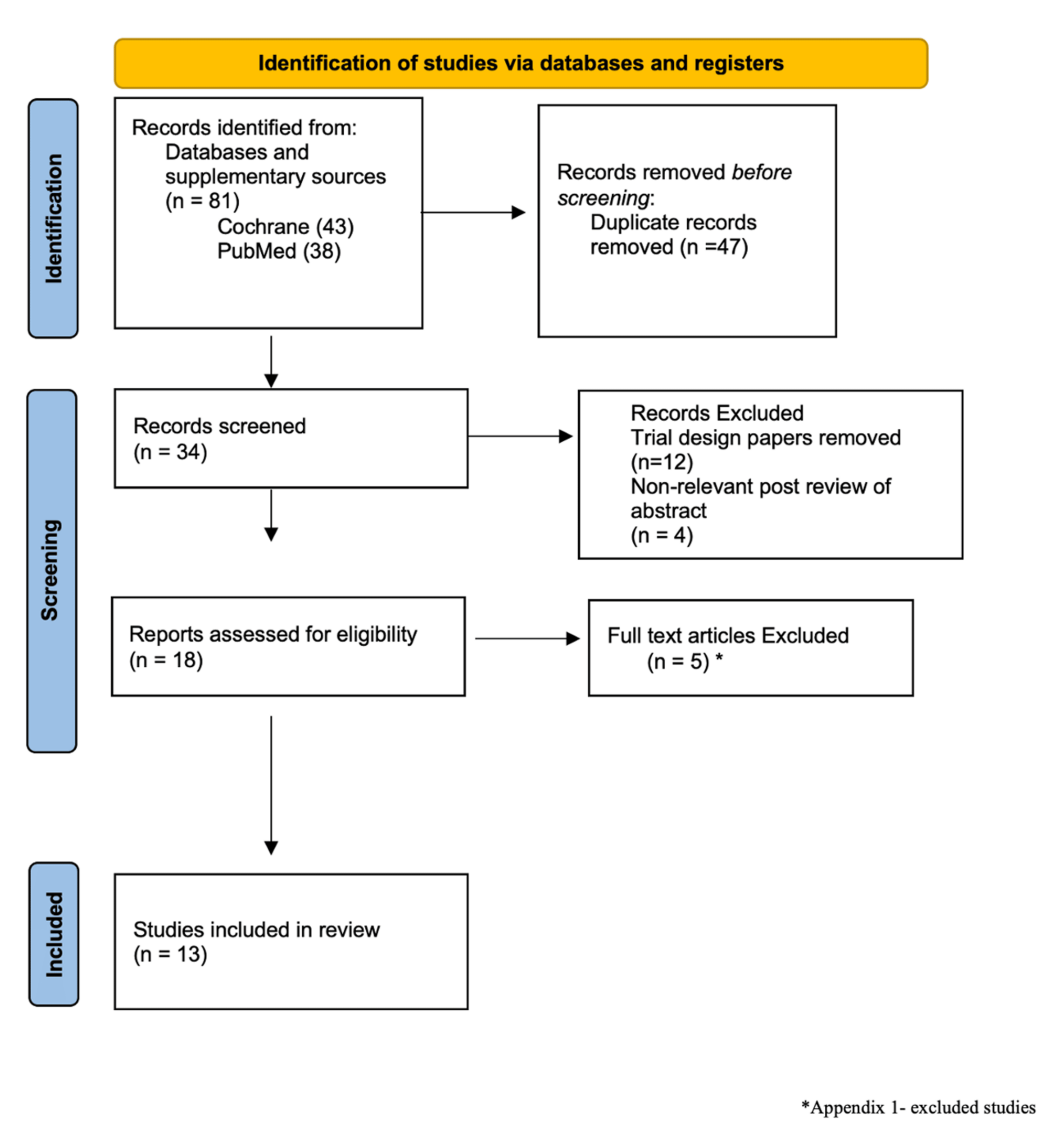
Preferred Reporting Items for Systematic Reviews and Meta-Analyses Extension for Scoping Reviews (PRISMA-ScR) flow diagram.

Figure 1 presents the Preferred Reporting Items for Systematic Reviews and Meta-Analyses Extension for Scoping Reviews (PRISMA-ScR) flow diagram, outlining the search strategy and study selection process in detail. Excluded studies and specific reasons for exclusion are listed in the Appendices.

Results

Summary of Evidence

A total of 13 peer-reviewed studies were included in this narrative review. The majority were prospective RCTs, with additional data from pilot studies, and a few multicenter or comparative cohort designs. These studies collectively assessed the clinical outcomes of TFL versus Ho:YAG in ureteroscopic lithotripsy, RIRS, and mini‑percutaneous nephrolithotomy (mini‑PCNL) procedures.

Efficacy (Stone-Free Rates)

SFR is a key indicator of treatment success in stone surgery. Across the included studies, TFL demonstrated either equivalent or superior SFRs compared to Ho:YAG. For example, Ulvik et al. reported a statistically significant improvement in SFR with TFL (92%) versus Ho:YAG (67%) (p = 0.001) in ureteroscopic treatment of ureteric and/or renal stones >5 mm [10]. Geavlete et al. similarly found higher SFRs with TFL (96.6% vs. 86.6%, p = 0.03) in patients undergoing RIRS [11]. Other studies, including Martov et al., Tg et al., Delbarre et al., and Chai et al., reported either superior or non-inferior outcomes with TFL [12-15]. However, it is important to note that these studies often involved mixed cohorts of ureteric and renal stones. This limits the ability to directly compare efficacy across individual surgical modalities.

*Operative* *Time*

TFL consistently demonstrated shorter operative durations across multiple studies. For instance, Chandramohan et al. reported mean operative times of 18.5 ± 1.5 minutes for TFL versus 31.6 ± 1.2 minutes for Ho:YAG (p < 0.05) [16]. Tg et al. found similar trends, with TFL procedures averaging 24.8 minutes compared to 27.3 minutes for Ho:YAG (p = 0.005) [13]. This reduction in operative time may translate into meaningful improvements in operating theater efficiency, especially relevant in healthcare systems with surgical backlogs.

Laser Performance and Ablation Efficiency

Multiple studies reported that TFL offers superior dusting performance and ablation speed. Taratkin et al. found that TFL’s use of smaller-caliber fibers (150 µm) led to better irrigation, more consistent lasering, and faster stone disintegration [17]. Although TFL devices are technically capable of operating at frequencies up to 2,400 Hz, typical clinical settings range from 50 to 500 Hz, depending on the procedure and stone characteristics. When combined with lower pulse energy, these settings enable fine fragmentation, which may reduce the need for basketing and shorten total lasering time. Taratkin et al. highlighted superior dusting performance, particularly with smaller-caliber fibers, enhancing irrigation and laser precision [17]. Vergamini et al. also reported higher surgeon satisfaction scores due to better maneuverability and reduced equipment noise during TFL procedures [18].

An important consideration when comparing laser platforms is the introduction of MOSES pulse modulation technology in Ho:YAG systems. MOSES modifies the laser pulse to create a vapor bubble that enhances energy delivery, leading to improved stone ablation and reduced retropulsion. Studies by Kudo et al. and Chai et al. have shown that although TFL generally outperforms conventional Ho:YAG in terms of ablation speed and operative efficiency, these advantages become less distinct, or even comparable, when Ho:YAG is used with MOSES technology [19,15]. In vitro and early clinical evidence suggest that MOSES-mode Ho:YAG significantly narrows the performance gap, particularly in stone clearance and procedure duration. Nonetheless, TFL maintains distinct advantages in fiber flexibility, irrigation flow, and dusting efficiency, which are particularly valuable in complex or anatomically restricted cases.

Retropulsion and Visibility

Reduced stone retropulsion was one of TFL’s most consistent advantages. Ulvik et al. and Taratkin et al. reported significantly lower stone migration rates in TFL groups, attributed to TFL’s lower peak power and high-frequency delivery [10,17]. These physical properties result in more stable lasering and minimize the need for stone repositioning. Enhanced visibility and scope deflection, enabled by thinner fibers, were also appreciated by surgeons, as reflected in improved satisfaction scores (e.g., Vergamini et al.) [18].

Safety and Complications

Both laser systems demonstrated favorable safety profiles. No study reported increased thermal injury or adverse outcomes with TFL. Ulvik et al. noted a lower rate of bleeding complications in the TFL group (5%) versus Ho:YAG (22%) (p = 0.014) [10]. Across all studies, the incidence of mucosal injury, ureteral perforation, and hematuria was low and comparable, affirming the safety of both technologies in experienced hands.

Cost Efficiency

Although TFL systems may incur higher initial capital costs compared to Ho:YAG, their operational efficiency may offset these expenses over time. Shorter operative times, reduced need for accessory devices such as stone baskets, and higher SFRs may translate into fewer retreatments and follow-up procedures, offering cumulative cost savings in high-volume settings [10]. TFL also supports high-frequency, low-energy dusting modes that produce fine particulate stone debris suitable for spontaneous clearance, often eliminating the need for basketing [19]. This not only reduces procedural complexity but may also shorten anesthesia time and improve patient comfort during recovery. These workflow efficiencies position TFL as a compelling option for healthcare systems such as the NHS, where throughput and cost-effectiveness are increasingly vital. Table 1 presents a summary of the included studies.

**Table 1:** Summary of the studies included in the review. Ho:YAG = Holmium:YAG; TFL = thulium fiber laser; SFR = stone-free rate

Study – first author (year)	Study type	Location of stones	SFR	Operative time	Laser efficiency/Ablation speed	Complications	Safety outcome
Ulvik et al. (2022) [10]	Prospective RCT	Ureteric + renal	Ho:YAG 67% vs TFL 92% (p = 0.001)	Ho:YAG 57 minutes vs. TFL 49 minutes (p = 0.008)	Not specified	Bleeding: 22% (Ho:YAG) vs. 5% (TFL) (p = 0.014)	Fewer intraoperative issues with TFL
Chandramohan et al. (2023) [16]	Prospective RCT	Ureteric	Equal SFR	Ho:YAG 31.6 ± 1.2 minutes vs. TFL 18.5 ± 1.5 minutes (p = 0.024)	Shorter lasering time; better ablation speed with TFL	Similar complication rates	Comparable safety profiles
Taratkin et al. (2023) [17]	Prospective RCT	Renal	Equal SFR	Ho:YAG 90.0 ± 31.8 minutes 200 µm SP vs. TFL 74.4 ± 26.3 minutes (significantly shorter than Ho:YAG, p = 0.044)	TFL used less energy; smaller fibers improved irrigation	Not detailed	Not detailed
Martov et al (2021) [12]	Prospective RCT	Ureteric	Equal SFR Ho:YAG 94.36% vs. TFL 100% (NS)	Ho:YAG group 24.7 ± 0.7 minutes vs. TFL 32.4 ± 0.7 minutes (p = 0.05)	TFL showed better ablation speed and less retropulsion	No significant difference	Safe for both modalities
Vergamini et al. (2023) [18]	Prospective RCT; ongoing - preliminary results	Ureteric	Pending	Similar total operative time	Not specified	Similar complication rates	Equivalent safety
Geavlete et al. ((2022) [11]	Prospective comparative cohort study	Renal	Ho:YAG 86.6% vs. TFL 96.6% (p = 0.03)	Not specified	TFL showed better ablation speed	No significant difference	Safe for both modalities
Gupta et al. (2025) [20]	Prospective RCT	Renal	Equal SFR (~82% vs. 79%)	Similar total operative time	Comparable efficiency and energy usage	Similar complication rates	Safe for both systems
Kaushik et al. (2025) [13]	Prospective RCT	Ureteric	Equal SFR	Ho:YAG 27.3 ± 2.77 minutes vs. TFL 24.8 ± 2.58 minutes (p = 0.005)	Significantly faster speed	No difference noted	Equivalent safety
Delbarre et al. (2023) [14]	Single-center cohort study	Ureteric and renal	Equal SFR Ho:YAG 68.4% vs. TFL 72% (NS)	Ho:YAG 21.4 minutes vs. TFL 19.9 minutes (NS)	Not specified	No difference noted	Equivalent safety
Haas et al. (2023) [21]	Prospective RCT	Ureteric and renal	Equal SFR	Ho:YAG 21.4 minutes vs. TFL 19.9 minutes (NS)	Not specified	No difference noted	Equivalent safety
Chai et al. (2024) [15]	Multicenter cohort Prospective Study	Renal + upper ureteric	Equal SFR	Similar total operative time	Not specified	Not stated	Reported safe
Gauhar et al. (2025) [22]	Propensity-matched comparative study	Renal	Equal SFR Ho:YAG 52.1 vs. TFL 64.6 (NS)	Similar total operative time	Not specified	No difference noted	Safe
Kudo et al. (2025) [19]	Retrospective single-center cohort study	Ureteric and renal	Equal SFR Ho:YAG 95.8% vs. TFL 97.9 (NS)	Similar total operative time	Not specified	Minimal	Safe

Discussion

This review synthesizes findings from 13 contemporary studies, including eight prospective RCTs, comparing TFL and Ho:YAG in the management of urinary stone disease. The data consistently demonstrate that TFL performs at least equivalently, and often superiorly, across key surgical metrics. SFRs were significantly higher in several studies evaluating TFL, most notably in Ulvik et al. (92% vs. 67%, p = 0.001) [10]. Other studies, including those by Geavlete et al., Kaushik et al., and Kudo et al., also reported enhanced efficacy [11,13,19]. Furthermore, operative efficiency was consistently improved with TFL, as studies by Chandramohan et al. and Martov et al. reported shorter operative durations and reduced intraoperative complexity [16,12].

The consistently high SFRs observed across multiple procedures suggest that TFL offers at least equivalent, and in many cases superior, clinical efficacy compared to Ho:YAG. In particular, the statistically significant improvement seen in ureteroscopic studies (e.g., Ulvik et al.) supports the potential of TFL to improve first-time clearance rates. While some RIRS studies showed more modest differences, the overall trend favored TFL or showed equivalence. These findings must be considered in light of patient selection, stone characteristics, imaging follow-up methods, and definitions of “stone-free,” which varied across studies and may influence outcomes. The improved outcomes are attributed to TFL’s ability to create finer dust particles, enhance irrigation and visibility through thinner fibers, and provide stable lasering with reduced retropulsion.

These findings are supported by the broader evidence base. A recent meta-analysis by Tang et al. (2024) pooled data from 13 studies (n = 1,394) and confirmed that TFL is associated with significantly higher SFRs, shorter operative times, and reduced stone migration compared to Ho:YAG, without an increase in complication rates [9]. Complementing this, Gan et al. (2025) conducted a comparative review of super-pulse TFL and Ho:YAG, reporting broadly similar SFRs and operative times but highlighting TFL’s clear advantages in terms of intraoperative visibility and reduced retropulsion [23]. Together, these systematic reviews reinforce the view that TFL not only meets but often exceeds Ho:YAG’s performance in key procedural domains.

It is important to recognize that the relative importance of laser characteristics varies across surgical settings. In ureteroscopy, where retropulsion can displace stones into the kidney, the lower pulse energy and smoother fragmentation of TFL help minimize migration and reduce the need for repositioning or basketing [19,20]. In RIRS, irrigation, visibility, and scope deflection are critical; the use of smaller TFL fibers (150-200 µm) improves irrigation flow and flexibility, enhancing access to calyceal stones and allowing finer dusting [10,17]. By contrast, mini‑PCNL prioritizes rapid fragment evacuation and procedural efficiency. Here, TFL’s ability to operate at very high frequencies with consistent dusting may reduce the need for basket retrieval and shorten operative times. These setting‑specific considerations illustrate that the advantages of TFL are not uniform but rather procedure‑dependent, aligning its physical properties with the demands of each surgical technique.

Importantly, these technical benefits have potential system-level implications. Within the NHS and other strained healthcare systems, the ability to reduce operative time could improve surgical throughput, alleviate waiting list pressures, and ultimately enhance access to care. The improved SFRs associated with TFL may also reduce the need for secondary interventions and postoperative imaging, translating into long-term savings.

Limitations

This review identifies several important limitations in the current body of evidence comparing TFL and Ho:YAG lithotripsy. A major issue is the predominance of single-center studies with relatively small sample sizes and short follow-up periods, typically fewer than three months. These factors limit the generalizability of findings and preclude assessment of long-term outcomes such as stricture formation, recurrence, and cost-effectiveness.

There is also considerable heterogeneity in intraoperative protocols. While many studies reported baseline laser settings, surgeon-led adjustments were commonly permitted, introducing variability in energy delivery and procedural efficiency. Similarly, fiber caliber varied between TFL and Ho:YAG systems, with TFL’s thinner fibers potentially conferring advantages in irrigation and scope deflection, yet no study employed validated tools to quantify intraoperative visibility or ergonomics.

A further limitation is that many studies included heterogeneous patient cohorts, incorporating both ureteric and renal stones, sometimes even mini-PCNL cases, without consistently stratifying outcomes by procedure type. As a result, while we attempted to categorize studies under ureteroscopy, RIRS, and mini-PCNL, wherever possible, the lack of procedure-specific subgroup reporting within individual studies reduces the precision of surgery-wise comparisons. This is especially important given that the efficacy, safety, and efficiency of laser platforms can vary substantially between these approaches.

Interpretation of SFR outcomes is influenced by variation in postoperative imaging methodology. The majority of included studies appropriately utilized non-contrast CT of the kidney, ureter, and bladder (KUB) for detecting residual fragments. However, a few studies, specifically Haas et al. and Delbarre et al [21,14], relied on less sensitive modalities such as ultrasound or plain KUB performed at one to three months postoperatively. These approaches could underestimate residual fragments and may falsely elevate reported SFRs. Notably, Geavlete et al. employed second-look ureteroscopy, which offers even greater sensitivity, although this method was not replicated in other trials [11]. This variation in imaging modality and follow-up timing complicates direct comparisons across studies and limits the reliability of pooled outcome interpretation.

Patient selection criteria further limit generalizability. Most trials enrolled individuals with stone burdens <2 cm, and few addressed outcomes in patients with more complex or larger calculi. The inconsistent use of ureteral access sheaths (UAS) may also impact outcomes such as retropulsion or access time, although large-scale registry data suggest UAS has minimal influence on SFR.

While capital costs for TFL systems remain high, their longer fiber durability, reduced need for disposable instruments such as baskets, and potential for shorter operative times could translate into long-term savings, particularly in high-volume or publicly funded healthcare systems. However, economic evaluations remain scarce and should be prioritized in future studies.

To build on current findings, larger multicenter RCTs with standardized laser protocols and extended follow-up are essential. These should include robust economic analyses and validated assessment tools for procedural visibility, surgeon ergonomics, and patient-reported outcomes. Such data will be critical to informing clinical guidelines, equipment procurement, and training strategies.

## Conclusions

While Ho:YAG remains the established global standard for laser lithotripsy, TFL demonstrates several compelling clinical and technical advantages that position it as a potential future leader in endourological laser lithotripsy. Its capacity for shorter operative times, reduced retropulsion, and superior ablation efficiency not only enhances surgical precision and throughput but may also offer tangible system-level benefits. In the context of increasing surgical demand and ongoing pressures on waiting lists, the improved procedural efficiency associated with TFL could contribute meaningfully to reducing operative backlogs and optimizing theater utilization. In addition to current evidence, a number of ongoing trials are expected to provide further insights into TFL’s long-term efficacy, safety, and cost-effectiveness. As these studies reach completion, they will be crucial in validating TFL’s emerging role and may inform future guidelines and broader adoption in endourological practice.

## Appendices

**Table 2:** Rationale for the studies screened but excluded.

Author (year)	Title	Reason for Exclusion
Ryan et al. (2022)	*Ureteroscopy with thulium fiber laser lithotripsy results in shorter operating times and large cost savings*	*Did not report stone-free rate (SFR) due to COVID-related follow-up gaps*
Singh et al. (2022)	*Thulium fiber LASER (TFL) vs Holmium LASER (HOL) for kidney stones in RIRS: a randomised controlled trial*	Conference abstract only – preliminary report without a full‑text peer‑reviewed manuscript or complete outcome data
Ya‑fei et al. (2020)	*Clinical **study on **efficacy and **safety of **thulium **laser in **treating **complex **urinary **calculi*	Non‑English full text (Chinese); English abstract insufficient for full data extraction
Ghazi et al. (2021)	*Ho:YAG versus thulium laser fiber for upper tract calculi: a clinical comparison*	Conference abstract (J Urol supplement); lacked full‑text peer‑reviewed article and methodological details
Popov et al. (2020)	*Comparison of the clinical efficacy of holmium and thulium ureterolithotripsy*	Non‑English (Russian) publication with abstract only; incomplete outcome reporting
